# Progress in respiratory management of bulbar complications of motor neuron disease/amyotrophic lateral sclerosis?

**DOI:** 10.1136/thoraxjnl-2016-208919

**Published:** 2016-08-12

**Authors:** Anita K Simonds

**Keywords:** Non invasive ventilation, Cough/Mechanisms/Pharmacology, Palliative Care

Loss of motor neurons in the cortex, brainstem and spinal cord is the hallmark of motor neuron disease/amyotrophic lateral sclerosis (MND/ALS), resulting in weakness of limbs, respiratory and bulbar muscles and eventually death from respiratory failure in the majority of patients. Around 20%–30% have bulbar symptoms at onset—this is less common in younger patients, but affects more than 40% of those over 70 years.^1^ Virtually all patients will develop bulbar symptoms with disease progression. Symptoms consist of dysarthria, dysphagia, difficulties clearing oropharyngeal secretions, impaired cough, choking, laryngospasm and aspiration. Each symptom is attributable to lower motor neuron weakness (true bulbar palsy), upper motor neuron weakness (pseudobulbar palsy) or a combination of both.

Safe swallowing requires adequate strength and coordination of orofacial and lingual muscles. With increasing weakness, difficulties in mastication, bolus preparation and oral food transport develop resulting in oral, pharyngeal and laryngeal residues of food. Lip closure and problems swallowing saliva lead to drooling. Failure of the larynx to move superiorly and anteriorly on swallowing and incomplete closure leads to laryngeal penetration or overt aspiration into the lungs.

The clearance of aspirated food, saliva or bronchial secretions depends on a forceful cough. An effective cough requires the inspiratory muscles to generate a deep inspiration of around 2–4 L, followed by closure of the glottis and the generation of a peak cough flow (PCF) of 360 to 1000 L/min in adults—depending on age, size and gender. This level of expiratory flow after glottal opening is required to shear secretions and debris from the airway and lift these to the upper airway/mouth. It is assumed that a minimum PCF of 160 L/min is required in adults and is estimated that those with values of 270 L/min or less may be prone to secretion retention at the time of chest infections, as these are associated with a decrease in respiratory muscle strength.^2^

Clearly, swallowing, breathing and coughing need to be integrated. In normal awake humans, swallows are accompanied by an apnoeic pause of 0.6–2.0 s duration and this apnoea is followed by expiration in more than 90% of swallows. Patients with MND/ALS tend to have a lower swallow volume and swallows may be followed by inspiration or a prolonged swallow apnoea. Those with pseudobulbar weakness have most disturbed respiratory/swallow coordination and aspiration is more likely if inspiration rather than expiration follows an ineffective swallow. Hadjikoutis and Wiles^1^ speculate that in a situation of mechanical loading of respiratory muscles, respiratory reflexes will prevail and/or loss of corticospinal fibres may decrease descending inhibition of respiration, thereby increasing the likelihood of inspiration after a swallow. Additionally, laryngospasm has been reported in up to 19% of patients with MND/ALS^3^ and nocturnal stridor has been reported in 13% of patients with multiple system atrophy. Laryngeal dysfunction can be caused by lower motor neuron failure of abductor muscles or upper motor neuron hyper-reactivity of adductor muscles and may occur early in the natural history before any other symptoms.^4^

The National Institute for Health and Care Excellence pathway for managing respiratory function in motor neuron disease^5^ suggests careful evaluation of respiratory and bulbar symptoms followed by measurement of SpO_2_ and FVC or sniff inspiratory pressure/maximum inspiratory pressure. It should be borne in mind that patients with bulbar and facial muscle weakness may not be able to use a mouthpiece effectively without leak and so measurement of spirometry may be underestimated. Voluntary effort is dependent on corticobulbar and corticospinal pathways which are frequently affected by MND/ALS, so in pseudobulbar patients the ability to carry out a forced manoeuvre, cough to command or to synchronise a spontaneous cough effort with manual assistance may be reduced and respiratory function underestimated—while reflex-induced cough may be preserved. Non-volitional tests such as measurement of diaphragmatic and oesophageal pressures during electrical or magnetic stimulation can distinguish between these aetiologies although these are relatively invasive.

Impairment of swallowing coordination and cough efficacy will predispose the individual to the risk of choking and pulmonary aspiration. Teaching the patient and family/carers physiotherapy techniques such as manually assisted cough and breath stacking using a lung volume recruitment bag (eg, ‘Ambu’ bag) with one way valve can be simple and inexpensive first steps.^6^ Mechanical insufflation-exsufflation (MI-E) has been used increasingly in individuals with neuromuscular weakness despite the lack of large randomised trials, although there is cumulative evidence that MI-E increases PCF and in combination with non-invasive ventilation (NIV) can prolong survival.^7^ ^8^ However, NIV and MI-E are reported to be less effective in those with bulbar involvement.^9^ Furthermore, it is often taught that patients with spastic upper motor neuron weakness are least likely to tolerate MI-E as upper airway collapse/spasm may be generated. This might be particularly the case at higher pressure spans which are reported to be most effective in other cohorts with neuromuscular disease.

In *Thorax* Andersen *et al*^10^ explored laryngeal and upper airway response patterns to MI-E in patients with bulbar MND/ALS to establish limiting factors and find out whether MI-E can be successfully applied. The authors carried out flexible transnasal fiberoptic laryngoscopy with video recording in a group of 20 patients with MND/ALS and 20 age-matched and gender-matched controls. Six patients had spinal MND with limb involvement but no bulbar symptoms, seven had hypotonic bulbar weakness and seven had pseudobulbar spastic bulbar features. Both groups with bulbar symptoms tended to have a lower PCF than those with a limb weakness presentation. Recordings used an MI-E protocol with insufflation pressures of 20–50 cm H_2_O. The key findings were that *adduction* of aryepiglottic folds occurred on insufflation in groups with hypotonic bulbar and spastic pseudobulbar palsy compared with healthy controls and those without bulbar symptoms. In addition, both true vocal cords and aryepiglottic folds tended to close during insufflation in pseudobulbar patients, particularly at high pressures. During exsufflation in all patients with MND/ALS there was *constriction* of the hypopharynx—an exaggeration of the finding seen in normal subjects to a varying degree. Constriction seemed very pronounced in those the hypotonic bulbar weakness (4/7 compared with 1/7 pseudobulbar patients). Clearly airway closure on both insufflation and exsufflation is a major limitation for applying MI-E and is likely to be distressing in those patients most severely affected.

A major limitation of the study is small number of patients studied which makes wider generalisation, statistical analysis and differentiation between findings in hypotonic bulbar and upper motor neuron bulbar patients especially difficult—an area where we critically need more information. However, a plausible if incomplete narrative emerges, which chimes with clinical impressions in many units.

A parallel experience with tolerance of devices has emerged from the use of NIV in MND/ALS. Aboussouan *et al*^11^ showed that in patients with MND/ALS having orthopnoea or PaCO_2_ >6.0 kPa, those who tolerated NIV had a longer survival than those who were unable to tolerate it and the group which tolerated NIV had a higher FVC and fewer bulbar symptoms. In a review^12^ of predictors of NIV tolerance, patients with limb onset were more like to be able to adhere to NIV compared with those with bulbar onset with an OR of 6.25 (95% CI 1.09 to 33.33). Age had no effect, but it is not obvious whether bulbar patients had more advanced disease. Farrero *et al*^13^ showed that the introduction of protocol-based NIV improved outcome particularly in non-bulbar patients. For the bulbar group, survival in those who tolerated NIV compared with those who were unable to did not differ, but for the subgroups who were hypercapnic, on initiation of NIV, survival was increased. In a landmark randomised trial of NIV in patients with MND/ALS, Bourke *et al*^14^ found a survival advantage in patients with mild or moderate bulbar weakness, but no increase in survival in those with severe bulbar involvement. Importantly, however, even severely affected bulbar patients had an improvement in sleep-related symptoms.

It should be recognised that extending survival with NIV in those with mild-to-moderate bulbar disease will over time lead to a greater prevalence of patients with severe bulbar disease. Furthermore, the presumption from early studies that patients with bulbar disease cannot tolerate NIV has been disproved. Farrero *et al*^13^ report that over 50% of bulbar patients were successfully initiated on NIV and the figure may be higher depending on the extent of bulbar problems. The authors attribute success to a less nihilistic attitude, inpatient initiation and overnight adaptation of settings.

So what have we learned? First, an understanding of the pathology and careful titration of settings make a difference. Andersen *et al*^10^ have shown that adduction of supraglottic structures limits insufflation in patients with bulbar MND/ALS. They surmise that upper airway reflexes may be hyper-regulated to prevent aspiration of foreign material into the airways which will stimulate glottis closure and that the collapse of more floppy supraglottic structures may occur as a result of the Bernoulli effect. Furthermore, pharyngeal construction occurs with exsufflation. The authors found it difficult to separate predominant problems in hypotonic bulbar palsy and hypertonic pseudobulbar patients, probably because of small numbers in the trial. However, higher pressures were most problematical and the team advise an individualised approach, starting with low insufflation pressures, with longer insufflation time, and gradually increasing these settings according to tolerance and efficacy. The addition of exsufflation may, or may not, be helpful. A similar approach is appropriate in starting NIV—high inspiratory pressures may generate airway closure and so pressure should be gently titrated upwards. Andersen *et al* suggest performing laryngoscopy if difficulties arise when determining MI-E settings, but this is unlikely to be available to all patients or feasible to repeat as the disease progresses, so clinical judgement and common sense are required. Crucially it should not be assumed that NIV and MI-E use in bulbar patients is futile—rather each case should managed individually, with the expectation that settings and interventions may need to be modified with disease progression.

Multidisciplinary input including speech and language therapy, physiotherapy and palliative care are important and improve outcome.^15^ Placement of a feeding gastrostomy and management of secretions is necessary to maintain nutrition and reduce risk of aspiration. A tracheostomy may be required in the presence of severe laryngeal spasm, or recurrent aspiration, but may not benefit quality of life if bulbar symptoms occur at end stage and if the care package consequences are significant. The advantages and disadvantages of performing a tracheostomy should be carefully explored as part of advance decision-making. No therapy or intervention, whether it is NIV, tracheostomy ventilation or MI-E, should be pursued if burdens outweigh benefit.

It is worth bearing in mind that all is not lost if MI-E and/or NIV cannot be initiated or maintained. This may not affect survival if the patient is normocapnic and other strategies can be considered, including manual physiotherapy techniques combined with palliative care. Ultimately the patients with bulbar MND/ALS do not fail MI-E or NIV—the therapy fails them and it is sensible that the terminology used to express this is both accurate and considerate.
